# Plant species phenology differs between climate and land‐use scenarios and relates to plant functional traits

**DOI:** 10.1002/ece3.11441

**Published:** 2024-05-23

**Authors:** Carolin Plos, Isabell Hensen, Lotte Korell, Harald Auge, Christine Römermann

**Affiliations:** ^1^ German Centre for Integrative Biodiversity Research (iDiv) Halle‐Jena‐Leipzig Leipzig Germany; ^2^ Institute of Biology, Geobotany and Botanical Garden, Martin Luther University Halle‐Wittenberg Halle (Saale) Germany; ^3^ Department of Community Ecology, Helmholtz‐Centre for Environmental Research (UFZ) Halle (Saale) Germany; ^4^ Institute of Ecology and Evolution with Herbarium Haussknecht and Botanical Garden, Friedrich Schiller University Jena Jena Germany

**Keywords:** climate change, flowering phenology, global change experimental facility (GCEF), grazing, mowing, species‐specific responses

## Abstract

Phenological shifts due to changing climate are often highly species and context specific. Land‐use practices such as mowing or grazing directly affect the phenology of grassland species, but it is unclear if plants are similarly affected by climate change in differently managed grassland systems such as meadows and pastures. Functional traits have a high potential to explain phenological shifts and might help to understand species‐specific and land‐use‐specific phenological responses to changes in climate. In the large‐scale field experiment *Global Change Experimental Facility* (GCEF), we monitored the first flowering day, last flowering day, flowering duration, and day of peak flowering, of 17 herbaceous grassland species under ambient and future climate conditions, comparing meadows and pastures. Both climate and land use impacted the flowering phenology of plant species in species‐specific ways. We did not find evidence for interacting effects of climate and land‐use type on plant phenology. However, the data indicate that microclimatic and microsite conditions on meadows and pastures were differently affected by future climate, making differential effects on meadows and pastures likely. Functional traits, including the phenological niche and grassland utilization indicator values, explained species‐specific phenological climate responses. Late flowering species and species with a low mowing tolerance advanced their flowering more strongly under future climate. Long flowering species and species following an acquisitive strategy (high specific leaf area, high mowing tolerance, and high forage value) advanced their flowering end more strongly and thus more strongly shortened their flowering under future climate. We associated these trait–response relationships primarily with a phenological drought escape during summer. Our results provide novel insights on how climate and land use impact the flowering phenology of grassland species and we highlight the role of functional traits in mediating phenological responses to climate.

## Graphical abstract

We investigated how future climate and land‐use type (i.e. mowing vs. grazing) individually and interactively affect the flowering phenology of grassland plant species in a large‐scale field experiment. Both, climate and land use impacted the flowering phenology of plant species in species‐specific ways. Functional traits related to the growth strategy and the phenological niche explained species‐specific phenological climate responses.

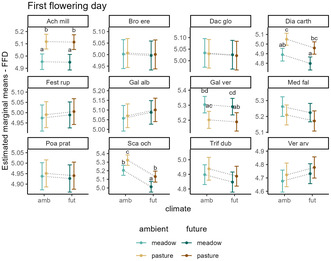

## 1 INTRODUCTION

Climate‐induced shifts in phenology are reported for a broad set of organisms with increasing temperatures mostly leading to advances of phenological events (Cleland et al., 2007; Parmesan, 2007; Root et al., 2003). Plants tend to show earlier spring phenology, often accompanied by a later autumn phenology, resulting in an extended growing season (Ahas et al., 2002; Badeck et al., 2004; Menzel et al., 2006; Menzel & Fabian, 1999). However, the direction and magnitude of phenological shifts are frequently reported to be species‐specific (Bock et al., 2014; Bucher et al., 2018; Jentsch et al., 2009; Piao et al., 2019; Root et al., 2003) and context‐specific. For example, phenological responses to changes in climate may depend on community composition (Jentsch et al., 2009), habitat type (König et al., 2018), observation site (Bucher et al., 2018), or growth form (Horbach et al., 2023; König et al., 2018). In addition to changes in temperature, changes in precipitation are identified as major drivers of phenological shifts (Jentsch et al., 2009; König et al., 2018; Lesica & Kittelson, 2010). Specifically, drought and heavy rain events can cause phenological shifts of the same magnitude as one decade of gradual warming, as shown in a global change experiment (Jentsch et al., 2009).

Shifts in flowering phenology are of special interest as they can impact biotic interactions like pollination or competition (Forrest & Miller‐Rushing, 2010; Gérard et al., 2020; Wolkovich & Cleland, 2011). While shifts in first flowering day are well studied (Bucher et al., 2018; Fitter et al., 1995; König et al., 2018), shifts in other phenological characteristics like last flowering day, flowering duration or peak flowering that strongly affect pollination and reproductive success, are less well studied but recently shown to also shift due to changes in climate (Bock et al., 2014; Bucher & Römermann, 2020; CaraDonna et al., 2014; Jentsch et al., 2009). Species‐specific changes in flowering phenology can lead to pollination mismatches when plants shift their flowering times while pollinators do not adapt, or vice versa (Forrest, 2015; Gérard et al., 2020; Hegland et al., 2009; Memmott et al., 2007), potentially affecting plant reproduction and pollinator fitness alike.

Grassland systems are among the most species‐rich habitats in Central Europe (Wilson et al., 2012) and are maintained by a long tradition of anthropogenic land‐use. Although mowing and grazing directly affect phenological dynamics of grassland species, studies on the effect of land‐use type on flowering phenology are scarce (but see Reisch & Poschlod, 2011; Reisch & Poschlod, 2009; Tadey, 2020; Völler et al., 2017; Völler et al., 2013). Plant species have been shown to adapt their flowering timing to typical land‐use times (Reisch & Poschlod, 2009; Völler et al., 2013), for example, plants growing on meadows flowered earlier compared to pastures (Reisch & Poschlod, 2009, 2011; Van Tienderen & van der Toorn, 1991). Mowing and grazing impose different disturbances to the vegetation and can differently affect plant growth performance (Brys et al., 2004; Herz et al., 2017; Römermann et al., 2009) and microclimate (Briemle et al., 2002; Zhu et al., 2016) on meadows and pastures. Selective grazing and small‐scale disturbances, due to trampling, affect the vegetation unevenly and create heterogeneous conditions in terms of light availability, open soil, small‐scale variation in (soil‐)temperature, and water balance through changes in soil pore volumes (Borer et al., 2014; Briemle et al., 2002; Lezama & Paruelo, 2016). In contrast, non‐selective mowing creates homogeneous conditions regarding light availability and mowing tractors can lead to a more uniform soil compaction (Chyba et al., 2014). Thus, microclimatic and microsite conditions can differ between mown and grazed sites (Zhu et al., 2016, shown for grazing exclusion) and changes in macroclimatic conditions (i.e., climate change) may therefore affect plant communities and their phenology differently on meadows and pastures. Furthermore, the management timing and frequency of extensively used grasslands usually differs between mown and grazed sites (Gilhaus et al., 2017), likely affecting plant phenology in different ways.

As described above, both climate warming and land‐use type separately influence the flowering phenology of grassland species. To our knowledge, there is no study on the interactive effects of both drivers on the flowering phenology of individual plant species (but see Tadey (2020) for the effects of grazing and climate). Understanding the interactive effects of different global change drivers on plant phenology is crucial to be able to predict phenological responses in natural systems, as combinations of different drivers can lead to diverse to responses (Cleland et al., 2006). Interactive effects of land‐use type and climate change on grassland species were already shown regarding the population growth rate of *Bromus erectus* in the context of the same experiment as this study (Global Change Experimental Facility, Lemmer et al., 2021) and regarding the relative growth rate in six grassland species across Germany (Bütof et al., 2012). We would expect that climate change will affect plant phenology differently on meadows and pastures due to potentially different microclimatic and microsite conditions.

Phenological responses to climate or other drivers are oftentimes highly species‐specific (Bucher et al., 2018; Fitter & Fitter, 2002; Menzel et al., 2006) and plant functional traits have high potential in explaining species‐specific phenological patterns and phenological responses (Bucher et al., 2018; Bucher & Römermann, 2020; Fitter & Fitter, 2002; Horbach et al., 2023; König et al., 2018; Sporbert et al., 2022; Sun & Frelich, 2011). For example, specific leaf area, which is related to productivity, competitive ability, and growth performance (Pérez‐Harguindeguy et al., 2016; Wright et al., 2004) was shown to explain shifts in the first flowering day (Bucher et al., 2018; König et al., 2018). Plant height that is linked to competitive ability and productivity (Gaudet & Keddy, 1988; Moles et al., 2009) is among the most important traits explaining variations in the flowering phenology of herbaceous plants in a botanical garden study (Sporbert et al., 2022) and is positively related to flowering start (Bolmgren & Cowan, 2008; Liu et al., 2021; Segrestin et al., 2020; Sun & Frelich, 2011). Furthermore, the phenological niche relates to the magnitude of phenological climate responses: Earlier flowering plants are repeatedly shown to advance their flowering time more strongly under changing climate conditions (Fitter & Fitter, 2002; Lesica & Kittelson, 2010; Menzel et al., 2006; Miller‐Rushing & Primack, 2008) though contrasting patterns are found as well (Bucher et al., 2018). Long flowering species change their flowering duration more strongly compared to short flowering species, shown along an elevational gradient (Bucher & Römermann, 2020). Plant traits like plant height, growth, and life form or growth rate relate to the plants' tolerance to mowing, grazing, or trampling and can be summarized with grassland utilization indicator values (Briemle et al., 2002). We propose that plants that are more tolerant toward mowing, grazing, or trampling might be less affected by the land management compared to more sensitive plants, which could in turn also affect the responses to future climate conditions. We use this set of commonly available functional traits to understand species‐specific phenological responses to future climate and under different land uses.

Here we use an experimental approach to understand the interacting effects of climate and land‐use type on plant phenology and its associations to plant traits. We monitor the flowering phenology of characteristic grassland species under ambient and future climate conditions growing in the two different land‐use types: extensively managed species‐rich meadows and pastures in the Global Change Experimental Facility in Germany (Schädler et al., 2019). In contrast to purely observational studies, this experiment allows us to unequivocally identify causal effects of manipulated climate and land use on plant phenology under the same set of background abiotic and biotic conditions.

More specifically, we ask the following questions:

1. How does the flowering phenology of different grassland species respond to (a) future climate and (b) different land‐use types (i.e., mowing or grazing) and (c) what is the interacting effect of climate and land‐use on the flowering phenology?
2. Can functional traits explain species‐specific and land‐use‐specific responses in phenology to changes in climate?

## 2 MATERIALS AND METHODS

### 2.1 The global change experimental facility (GCEF)

Data were sampled in 2020 in the “global change experimental facility” (GCEF), a large‐scale field experiment to investigate the consequences of climate and land‐use change on ecosystem processes, located in Bad Lauchstädt in central Germany (51°22,060 N, 11°50,060 E, 118 m a.s.l.). The mean annual temperature of the study site is 9.7°C, the mean annual precipitation is 525 mm and the soil is a nutrient‐rich haplic chernozem (Schädler et al., 2019). The experiment is set up using a split‐plot design combining a climate treatment (ambient and future climate, abbreviated amb; fut) at the main‐plot level and different land‐use types at the sub‐plot level. The experiment was established in 2014. For detailed information on the experimental setup see Schädler et al. (2019) and for a schematic overview of the setup see Appendix S2 in Schädler et al. (2019). The future climate treatment was established according to projections of regional climate change models for 2070–2100, which corresponds with a realistic manipulation of future climate (Korell et al., 2020). This applies in particular to the precipitation manipulation: the spring and autumn precipitation is increased by ~10% using sprinkler systems and the summer precipitation is reduced by ~20% (see also Appendix S1, Figure S1) using movable roof systems. As temperature is passively manipulated by closing these roofs at night, the mean daily increase in temperature by ~0.55°C is less than projected (~2°C), but still leads to an increase in the number of growing degree days (GDD) by ~5.2% (Schädler et al., 2019). Minimum temperatures increased more strongly under the climate treatment (by ~1°C) than mean temperatures did (for more details see Schädler et al., 2019). According to the application of a whole scenario of future climate, the effects of altered precipitation patterns and altered temperature cannot be disentangled.

All observations were done on the two land‐use types extensively used meadows (abbreviated EM) and extensively used pastures (abbreviated EP). In the study year 2020, mowing was conducted once in the beginning of June (calendar week 24). Grazing occurred twice per year, in late May (calendar week 20 or 21) and late June (calendar week 26 or 27), as a high‐intensity, short‐time grazing with a group of ~20 sheep that remain on the pasture plots for 24 h. Because of the low overall productivity due to the extreme drought conditions during the years before (2018/19) there was a lower overall management intensity. Before 2018, management intensity was higher (usually three times grazing, two times mowing). Treatment plots (i.e., ambient meadows (EM amb), future meadows (EM fut), ambient pastures (EP amb), future pastures (EP fut)) are replicated five times leading to overall 20 experimental plots in this study. Each treatment plot has a size of 16 m × 24 m. In the center of each treatment plot a 3 m × 3 m permanent plot (hereafter referred to as “plot”) was established for the phenological observations. For more detailed information on the experimental setup and manipulation in the GCEF see Schädler et al. (2019).

### 2.2 Phenological monitoring

The flowering phenology (i.e., the presence of flowers (y/n)) and the flowering intensity (0%–100%) of each of the co‐occurring plant species in the plot were monitored once per week following the PhenObs protocol (Nordt et al., 2021). Per species, all individuals growing inside the plot were observed as a “population.” The phenological observations were conducted between 01 April 2020 and 04 December 2020. From the data, we extracted the day of the year (doy) for the phenological stages first flowering day (FFD), last flowering day (LFD), day of maximum flowering intensity resp. peak flowering (PeakFl) as well as the flowering duration (FD) (i.e., the number of days between FFD and LFD).

### 2.3 Species selection

Plant species that occurred and flowered in at least three out of five plots of each treatment combination (ambient meadow, future meadow, ambient pasture, future pasture) were selected from the monitoring data (see above “*Phenological monitoring*”) and used for further analysis. Thus, 17 of 95 species that occurred at the study site were selected (Table 1), from which four species were grasses, two legumes, and 11 herbs.

**TABLE 1:** Overview of plant species that occurred and flowered in all treatments together with mean trait values derived from (a) the observed phenological data (timing and length of phenological niche), (b) the TRY database (plant height, SLA) (Kattge et al., 2020), and c) BiolFlor (mowing, grazing, and trampling tolerance and forage value) (Klotz et al., 2002).

Species	Family	Timing of phenological niche	Length of phenological niche	Plant height	SLA	Mowing tolerance	Grazing tolerance	Trampling tolerance	Forage value
*Achillea millefolium* L.	Asteraceae	154	134	35	16.8	NA	NA	NA	NA
*Bromus erectus* Huds.	Poaceae	149	6	70	16.1	5	4	4	6
*Capsella bursa‐pastoris* (L.) Medik.	Brassicaceae	101	25	33	40.8	3	7	6	2
*Dactylis glomerata* L.	Poaceae	153	6	72	24.8	8	4	6	8
*Dianthus carthusianorum* L.	Caryophyllaceae	145	167	44	17.7	3	4	4	3
*Festuca rupicola* Heuff.	Poaceae	146	13	29	24.1	6	4	4	4
*Galium album* Mill.	Rubiaceae	158	117	60	23.2	7	3	3	4
*Galium verum* L.	Rubiaceae	191	84	45	17.7	5	4	4	4
*Medicago falcata* L.	Fabaceae	188	48	51	20.5	5	2	2	7
*Poa pratensis agg*.	Poaceae	140	9	47	20.2	8	8	8	6
*Scabiosa ochroleuca* L.	Dipsacaceae	193	115	41	11.6	5	3	4	4
*Senecio vulgaris* L.	Asteraceae	98	24	33	27.6	3	5	3	2
*Stellaria media* (L.) Vill.	Caryophyllaceae	94	20	32	42.2	7	4	4	3
*Taraxacum sect. Ruderalia* Kirschner et al.	Asteraceae	96	28	28	32.8	8	7	7	7
*Trifolium dubium* Sibth.	Fabaceae	138	19	25	29.4	7	4	4	7
*Veronica arvensis* L.	Scrophulariaceae	109	13	17	27.7	7	4	4	2
*Veronica persica* Poir.	Scrophulariaceae	97	20	21	35.0	NA	NA	NA	NA

*Note*: Abbreviations and units: Timing of phenological niche (first flowering day as day of the year ‐ doy), length of phenological niche (flowering duration in days), plant height (vegetative height in cm), SLA (specific leaf area in mm^2^/mg), mowing, grazing, trampling tolerance, and forage value are given in classes from 1 to 9 (from low to high tolerance or forage value) (Briemle et al., 2002). Nomenclature follows (Jäger, 2016).

Five of the 17 selected species (i.e., *Capsella bursa‐pastoris, Senecio vulgaris, Stellaria media, Taraxacum officinalis, Veronica persica*) already started flowering in some of the plots when the phenological monitoring was started and was thus excluded from the analysis of FFD and FD.

### 2.4 Functional trait data

To analyze whether functional traits can explain species‐specific and land‐use‐specific responses in phenology to changes in climate, trait data on various traits previously shown to be relevant to phenological patterns was compiled. Specific leaf area (SLA) and vegetative plant height were extracted from the TRY database (Kattge et al., 2020) and the mean value for SLA and plant height were calculated per species (Table 1). Grassland utilization indicator values (hereafter referred to as “grassland indicator values”) for mowing, grazing, trampling tolerance, and forage value are individual, morphological‐ecophysiological traits that were developed by experts (Briemle et al., 2002). They range between 1 and 9 and characterize plants according to their realized ecological niche regarding mowing, grazing, and trampling tolerance and evaluate the forage value for livestock (Briemle et al., 2002). More precisely, an indicator value of 1 represents species that do not tolerate mowing, grazing, or trampling, respectively, while a value of 9 represents a very high tolerance to the respective disturbance. They were developed and validated on decade‐long experience in grassland habitats taking life and growth form and plant height into account summarizing a suite of relevant traits (Briemle et al., 2002). Grassland indicator values from Briemle et al. (2002) were extracted from the BiolFlor database (Klotz et al., 2002) for all species for which information was available (*n* = 15, Table 1). For *Scabiosa ochroleuca* L., values of the closely related species *Scabiosa columbaria* L., that occurs in the same habitat, were used and for *Festuca rupicola* Heuff., values of the closely related species *Festuca ovina* L. s. str. that forms a species aggregate to which *F. rupicola* belongs, were taken.

To classify the phenological niche of the species as a species trait (i.e., early‐ and late‐flowering and short‐ and long‐flowering species), per species the mean first flowering day (FFD) and mean flowering duration (FD) were extracted from the control plots (ambient meadows and ambient pastures) and considered as additional functional traits. As a result, a trait table with a mean value per trait and species was used for further analysis (see Table 1).

### 2.5 Statistical analysis

All statistical analyses were performed in R Version 4.2.1 (R Core Team, 2022).

### 2.6 Main and interactive effects of climate and land use on plant phenology

To test for the effect of (a) climate (ambient vs. future), (b) land‐use type (meadow vs. pasture), (c) species, and (d) their interactions on phenology, generalized linear mixed effect models using the function *glmer* (family “Poisson”) from the package *lme4* (Bates et al., 2015, p. 4) were performed. As our phenological data were integer, never negative, had a left‐skewed distribution, and can be considered count data (number of days), we chose the family Poisson for the models. Each model was tested for overdispersion using the function *dispersion_glmer* from the package *blmeco* (Korner‐Nievergelt et al., 2015). Overdispersion was only detected for the model for flowering duration (FD), for which then the model was refitted using negative binomial distribution (function *glmmTMB*, package *glmmTMB*; Brooks et al., 2017). The day of the year (doy) for the phenological stages first flowering day (FFD), last flowering day (LFD), the day of peak flowering (PeakFl), and the flowering duration (FD, measured in number of days) served as response variables. As the GCEF is set up as a split‐plot design, main plot (i.e., experimental unit, *n* = 10, Schädler et al., 2019) nested in climate treatment (ambient or future) was used as random effect (1|mainplot:climate). The 17 species were present within land‐use sub‐plots rather than randomly assigned to separate experimental units, thus we considered them as the sub‐sub‐plot level. In order to avoid pseudo‐replication at the sub‐plot level (i.e., land use), we therefore included the interaction between land use and main plot nested in climate treatment as a second random effect (1|landuse:mainplot:climate). The models were simplified by stepwise removing non‐significant interaction terms in accordance with the AIC until the most parsimonious model was found. Estimated marginal means were calculated from the simplified models using the *emmeans* function from the package *emmeans* (Lenth, 2022) to identify significant differences between the treatment combinations for each plant species.

### 2.7 Explaining phenological shifts by functional traits

To quantify the climate effects on each phenological parameter (i.e., FFD, LFD, FD, Peakfl) for each land‐use type in a standardized way, log response ratios (LRR) were calculated for each species following Hedges et al. (1999). The LRR is calculated as the natural logarithm (ln) of the response ratio (RR) that is characterized as the quotient of the mean of the treatment group *x¯*_*T*_ (future climate) and the mean of the control group *x¯*_*C*_ (ambient climate), Equation 1:

(1) $$LRR=\ln\left({\bar{x}}_{\text{T}}/{\bar{x}}_{\text{C}}\right)=\ln\left({\bar{x}}_{\text{future}}/{\bar{x}}_{\text{ambient}}\right)$$

To test whether shifts in phenological stages (i.e., climate responses represented by LRRs) can be explained by plant functional traits (Table 1) and depending on the land‐use type, we performed linear models. As the indicator values for trampling and grazing tolerance were strongly correlated (*r* = .86, *p* < .001, Appendix S1, Figure S2) and as on short‐term intensively grazed pastures, grazing tolerance is equivalent to mowing tolerance combined with trampling tolerance, only trampling tolerance was considered for the models. The plant functional traits mean FFD, mean FD, SLA, plant height, mowing and trampling tolerance, and forage value, all alone and in interaction with land‐use type, served as explanatory variables (full model). The LRR of each phenological stage (i.e., LRR_FFD_, LRR_LFD_, LRR_FD_, LRR_PeakFl_) served as response variable, respectively. To identify the most parsimonious model identifying relevant traits for the four studied response variables we used the *dredge* function from the *MuMIn* package that selects the best model according to the Akaike information criterion (AIC) (Bartoń, 2023). The respective model selection tables are presented in Appendix S2, Tables S1–S4.

## 3 RESULTS

### 3.1 Main and interactive effects of climate and land use on plant phenology

**FIGURE 1:**
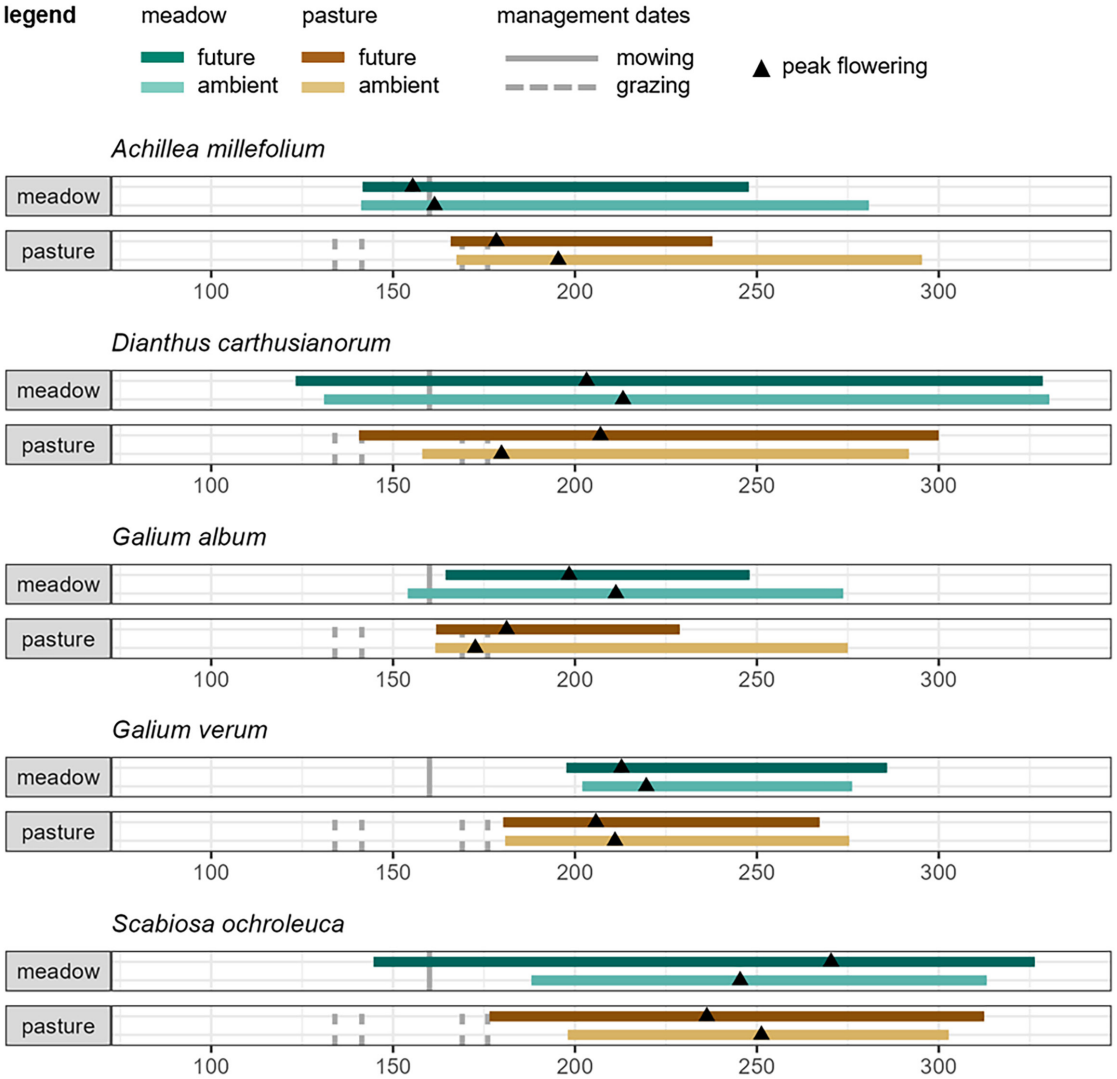
Flowering times and timing of land management (mowing or grazing) across all treatments for five exemplary species. Strips summarize the mean FFD, LFD, and day of peak flowering per treatment, the x‐axis shows day of the year (doy). The same figure on the remaining 12 species investigated can be found in Appendix S1, Figure S3.

Both climate and land use had significant effects on flowering phenology in a species‐specific way (Table 2). We did not find evidence for interactive effects of climate and land use on the flowering phenology, meaning that climate effects were independent of land‐use type (Table 2). Figure 1 gives an overview of the flowering times (i.e., start, peak, and end of flowering) per treatment (ambient meadow, future meadow, ambient pasture, future pasture) along with the timing of land‐use activities (mowing or grazing) exemplary for five species showing phenological responses to climate and land use. The same figures for all remaining species can be found in Appendix S1, Figure S3.

**TABLE 2:** Results of the simplified generalized linear mixed effect models testing for the effect of climate, land‐use, species, and their interactions on the flowering phenology, that is, first flowering day (FFD), last flowering day (LFD), flowering duration (FD), day of peak flowering (Peak flowering). Significant p‐values are highlighted in bold. Missing information refers to interaction terms that were excluded due to model simplification.

	FFD			LFD			FD			Peak flowering		
	*R* ^2^_marg._ = 0.76			*R* ^2^_marg._ = 0.95			*R* ^2^_marg._ = 0.82			*R* ^2^_marg._ = 0.92		
	*R* ^2^_cond._ = 0.76			*R* ^2^_cond._ = 0.96			*R* ^2^_cond._ = 0.83			*R* ^2^_cond._ = 0.92		

Predictors	Chi2	*df*	*p*‐value	Chi2	*df*	*p*‐value	Chi2	*df*	*p*‐value	Chi2	*df*	*p*‐value
Intercept	23401.7	1	**<.001**	39722.2	1	**<.001**	1152.6	1	**<.001**	39420.2	1	**<.001**
Climate	0.01	1	.91	23.37	1	**<.001**	0.18	1	.67	0.41	1	0.52
Land‐use	20.19	1	**<.001**	0.08	1	.78.	1.55	1	.21	23.47	1	**<.001**
Species	307.8	11	**<.001**	3116.1	16	**<.001**	957.4	11	**<.001**	2118.8	16	**<.001**
Climate* land‐use	–	–	–	–	–	–				–	–	–
Climate* species	32.7	11	**<.001**	66.27	16	**<.001**				–	–	–
Land‐use* species	54.5	11	**<.001**	38.41	16	**<.01**				57.3	16	**<.001**
Climate* land‐use* species	–	–	–	–	–	–				–	–	–

**FIGURE 2:**
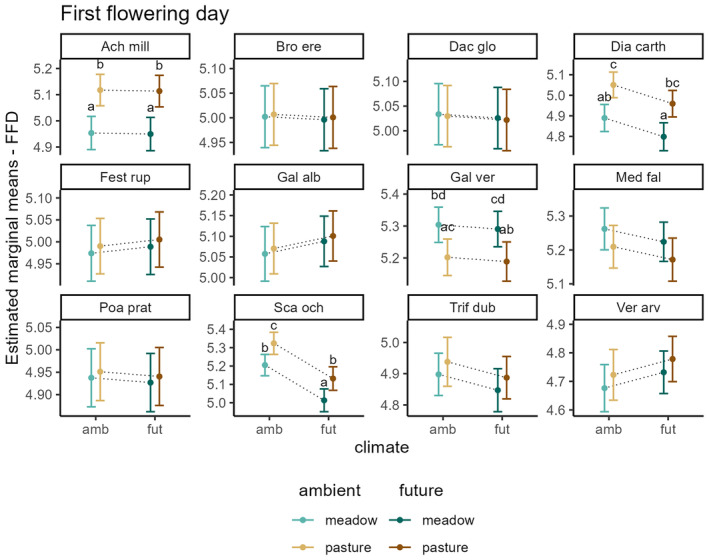
Effects of climate and land use on first flowering day. Shown are estimated marginal means of FFD with the 95% confidence intervals, resulting from the generalized linear mixed model. Results compare FFD between ambient and future climate on meadows and pastures for each species. Letters indicate significant differences between treatments. If no letters are shown, no significant difference between treatment groups was found. Dotted lines are only shown for a better interpretation of the interacting effects of climate and land use.

Regarding the first flowering day, the fixed effects explained 76% of the variation (*R* ^2^_marginal_ = .76). Land use and climate both affected the first flowering day in a species‐specific manner (significant species*climate and species*land‐use interactions, Table 2), however, there was no interaction between climate and land‐use. Figure 2 shows an overview of the species‐wise estimated marginal means of FFD with the 95% confidence intervals, resulting from the generalized linear mixed model. For example, the first flowering day of *Achillea millefolium* occurred significantly later in pastures than in meadows, while climate treatment had no effect (land‐use effect). In *Dianthus carthusianorum*, flowering started later in pastures than in meadows (land‐use effect) and tended to start earlier under future climate in both land‐use types (climate trend). In contrast, *Galium verum* flowered earlier in pastures than in meadows and tended to advance flowering under future climate. *S. ochroleuca* started flowering significantly later in pastures than in meadows and significantly advanced flowering under future climate in both land‐use types (climate and land‐use effect). *Galium album* and *Veronica arvensis* showed trends for delayed flowering under future climate, while *Medicago falcata* and *Trifolium dubium* showed trends for advanced flowering under future climate, although these trends were not significant. FFD of the other species did not significantly differ between land‐use and climate treatments.

**FIGURE 3:**
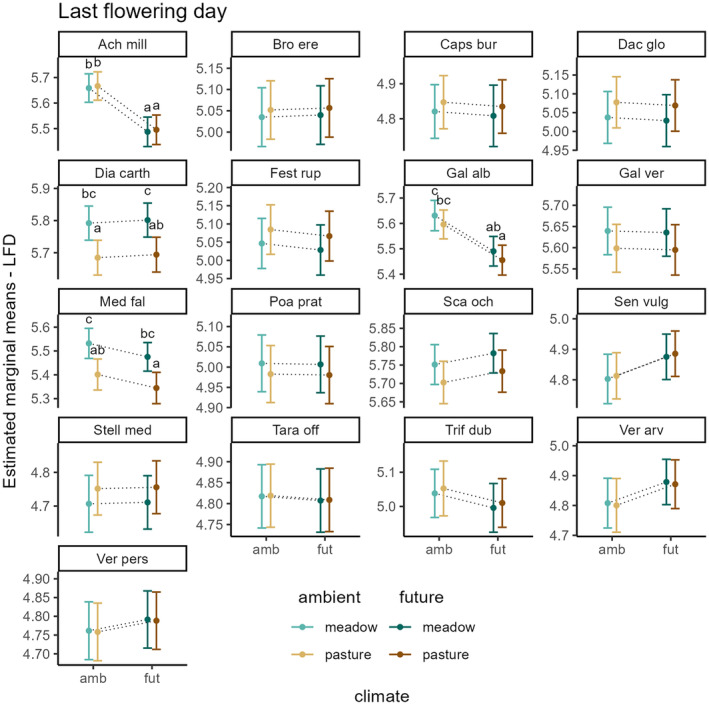
Effects of climate and land use on last flowering day. Shown are estimated marginal means of LFD with the 95% confidence intervals, revealed from the generalized linear mixed model. Results compare LFD between ambient and future climate on meadows and pastures for each species. Letters indicate significant differences between treatments. If no letters are shown, no significant difference between treatment groups was found. Dotted lines are only shown for better interpretation of the interactive effects of climate and land use.

For the last flowering day, the model explained 95% of the variation (*R* ^2^_marginal_ = .95). Similar to the first flowering day, species responded species‐specifically to climate and land use (Table 2), and climate effects were again independent of land‐use type. The following species responses are presented in Figure 3: We found significant treatment effects in four species. *A. millefolium* and *G. album* ended flowering significantly earlier under future climate regardless of land‐use type (climate effect). LFD of *D. carthusianorum* and *M. falcata* occurred significantly earlier on pastures (land‐use effect). Furthermore, under future climate LFD of *M. falcata* tended to advance while for *D. carthusianorum* LFD tended to delay (climate trends). LFD of the other species did not significantly differ between land‐use and climate treatments.

For flowering duration, the model explained 82% of the variation (*R* ^2^_marginal_ = .82). The investigated species differed in their flowering duration (species effect), but no significant effect of climate or land use was detected for the flowering duration (Table 2). See Appendix S1, Figure S4 for individual trends.

For the day of peak flowering, the model explained 92% of the variation (*R* ^2^_maginal_ = 0.92). Land use affected the day of peak flowering in a species‐specific manner (Table 2, Appendix S1, Figure S5), while climate did not have a significant effect. We found significant land‐use effects in two species. Peak flowering of *A. millefolium* occurred significantly later in pastures than in meadows regardless of climate (Appendix S1, Figure S5). In contrast, peak flowering of *G. album* occurred significantly earlier in pastures than in meadows (Appendix S1, Figure S5). Most other species showed similar trends either advancing or delaying peak flowering in pastures compared to meadows (Appendix S1, Figure S5).

### 3.2 Explaining phenological shifts by functional traits

**FIGURE 4:**
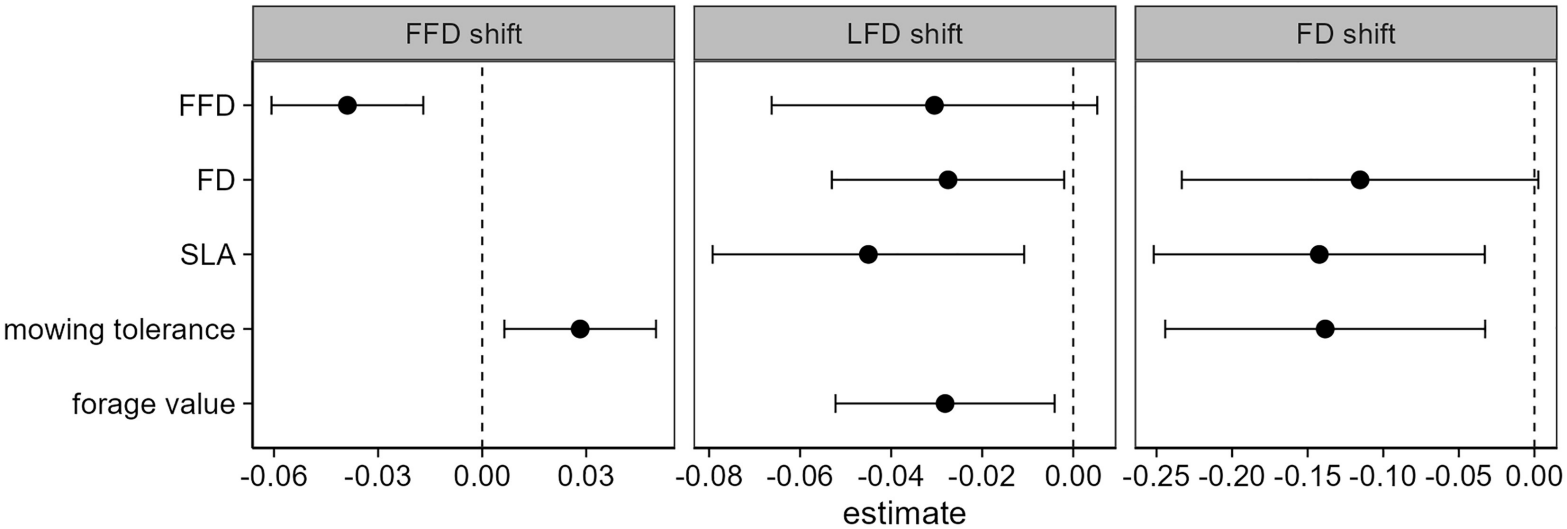
Effect of functional traits on the response to climate (LRR) of first flowering day (FFD shift), last flowering day (LFD shift), and flowering duration (FD shift). Estimates of the final linear models with 95% confidence intervals are shown. Abbreviations of response variables: FD, length of phenological niche measured as flowering duration; FFD, phenological niche measured as flowering start; SLA, specific leaf area.

Functional traits explained species‐specific phenological responses to changes in climate (LRR) (Figure 4, Table 3).

**TABLE 3:** Model results for the most parsimonious models explaining phenological shifts in first flowering day (FFD shift), last flowering day (LFD shift), and flowering duration (FD shift) to future climate by functional traits and grassland indicator values. None of the investigated traits related to shifts in peak flowering.

	FFD shift	LFD shift	FD shift
	*R* ^2^_multiple_ = 0.42 *R* ^2^_adjusted_ = 0.38	*R* ^2^_multiple_ = 0.36 *R* ^2^_adjusted_ = 0.26	*R* ^2^_multiple_ = 0.32 *R* ^2^_adjusted_ = 0.24
	*F*_2,27_ = 9.81; *p* < .001	*F*_4,25_ = 3.558; *p* = .019	*F*_3,23_ = 4.12; *p* = .016

Predictor	Estimate	SE	*p*‐value	Estimate	SE	*p*‐value	Estimate	SE	*p*‐value
Intercept	−0.013	0.011	.251	−0.004	0.009	.641	−0.022	0.048	.653
FFD	−0.039	0.011	.001**	−0.030	0.018	.107			
FD				−0.027	0.013	.044*	−0.115	0.060	.066.
SLA				−0.045	0.017	.016*	−0.142	0.055	.017*
Mowing tolerance	0.028	0.011	.017*				−0.138	0.054	.016*
Forage value				−0.028	0.012	.030*			

Abbreviations: FD, length of phenological niche measured as flowering duration in days; FFD, timing of phenological niche measured as first flowering day; SE, standard error; SLA, specific leaf area. Significance levels: *p < .05, **p < .01.

The best model describing changes in FFD contained the phenological niche (mean FFD) and the mowing tolerance (Table 3). The model selection table that displays all models with a delta AIC < 2 is shown in Appendix S2, Table S1. The model on shifts in FFD explained 42% of the variation (*R* ^2^_multiple_ = .42, *F*_2,27_ = 9.81, *p* < .001). The phenological niche (represented by the mean FFD under ambient climate) was negatively related to changes in FFD under future climate (Table 3). Late flowering species showed stronger advances in their first flowering day under future climate (Figure 4, Appendix S1, Figure S6). Mowing tolerance was positively related to the FFD response (Figure 4, Appendix S1, Figure S6). Species with a low mowing tolerance more strongly advanced FFD under future climate compared to species with high mowing tolerance, which tended to show no response (Appendix S1, Figure S6).

The best model describing changes in LFD contained the phenological niche (mean FFD), the length of the phenological niche (mean FD), SLA, and forage value (Table 3). The model selection table that displays all models with delta AIC < 2 is shown in Appendix S2, Table S2. The model on shifts in LFD explained 36% of the variation (*R* ^2^_multiple_ = .36, *F*_4,25_ = 3.56, *p* < .05, S2, Table 3). Long flowering species, species with a high SLA and high forage value advanced their LFD more strongly under future climate (Figure 4, Appendix S1, Figure S7). Late flowering species tended to advance LFD more strongly under future climate, even though this effect was non‐significant (*p* = .11, Figure 4, Appendix S1, Figure S7).

The best model describing changes in FD contained the length of the phenological niche (mean FD), SLA, and mowing tolerance (Table 3). The model selection table that displays all models with delta AIC < 2 is shown in Appendix S2, Table S3. The model on shifts in FD explained 32% of the variation (*R* ^2^_multiple_ = .32, *F*_3,23_ = 4.12, *p* < .05; Table 3). Shifts in the flowering duration (LRR_FD_) related to future climate were explained by SLA and mowing tolerance (Figure 4). Under future climate species with a high SLA and a high mowing tolerance shortened their flowering duration more strongly compared to species with low SLA and low mowing tolerance (Appendix S1, Figure S8). Furthermore, long flowering species tended to shorten their FD more strongly under future climate when compared to short flowering species, even though this effect was non‐significant (*p* = .06, Figure 4, Appendix S1, Figure S8).

The best model describing changes in peak flowering contained none of the variables, that is, none of the considered traits related to shifts in peak flowering. Thus, no further results can be reported. The model selection table that displays all models with delta AIC < 2 is shown in Appendix S2, Table S4.

The functional traits “plant height” and “trampling tolerance” did not relate to shifts in any of the tested phenological parameters. However, there is some indication that plant height might play a role for responses in LFD (compare model selection table in Appendix S2, Table S2) and that trampling tolerance might play a role for responses in FFD (model selection table in Appendix S2, Table S1).

Further, we did not find evidence for interactive effects between traits and land‐use type, meaning that the investigated traits were not of different importance for phenological shifts on meadows and pastures.

## 4 DISCUSSION

Our results clearly show that the flowering phenology of the 17 studied grassland species responded to both changes in climate and different land‐use types in species‐specific ways. Especially FFD and LFD were species‐specifically affected by climate and land use. Plant functional traits and grassland indicator values explained these species‐specific climate responses. Furthermore, different sets of traits explained shifts in the different phenological stages.

Conducting our study at the Global Change Experimental Facility enabled us to unequivocally identify the causal effects of manipulated climate and land use on plant phenology under the same abiotic and biotic background conditions. However, large experiments as this have the disadvantage of a smaller sample size (*n* = 5, i.e., five replicates per treatment) and the high variability of the data may mask subtle effects of climate or land use, thereby decreasing the likelihood to detect small but true effects (i.e., decreasing the statistical power). Therefore, in the following, we also present and discuss marginal effects and trends.

### 4.1 Effects of land use and climate on plant phenology

Land‐use type significantly influenced the flowering phenology in a species‐specific way. Most of the species growing on meadows flowered earlier than those growing on pastures, which is a frequently reported pattern (Reisch & Poschlod, 2009, 2011; Van Tienderen & van der Toorn, 1991), but we also found opposing patterns in single species. Phenological studies on the effects of land‐use type are scarce and usually link their findings to genetic differentiation and evolutionary processes that act on a larger temporal scale (Reisch & Poschlod, 2009; Völler et al., 2013, 2017). However, rapid responses of the flowering time to land use or other drivers such as climate have also been shown (Bucharova et al., 2024; Franks et al., 2007; Rauschkolb et al., 2022; Zopfi, 1993). Due to the comparably short duration of the experiment (6 years by the time of our study), we think that genetic differentiation or evolutionary processes have not yet played a prominent role in our study system, at least not for the perennial species strongly dominating our dataset. We assume, that the effects of the land‐use type alone are on the one hand attributed to the timing of the land management that differed between pastures and meadows (first grazing occurred 3–4 weeks before mowing). On the other hand, the heterogeneity of microsite conditions in meadows and pastures may also give rise to land‐use‐specific phenological patterns. For example, LFD of *D. carthusianorum* occurred significantly earlier on pastures than on meadows but long after the land management events. We observed higher proportions of open soil (Appendix S1, Figure S9) and increased maximum temperatures on pastures (Appendix S1, Figure S11), especially during summer, which probably led to generally more stressful growing conditions on pastures. Different land‐use types may furthermore alter biotic interactions like competition or herbivory that might further influence phenological patterns (Busch et al., 2018; Freeman et al., 2003; Gossner et al., 2014; Tadey, 2020; Völler et al., 2017). Overall, results from this study indicate that land‐use effects on the flowering phenology of grassland species are due to a combination of timing, frequency, altered microsite conditions, and biotic interactions on meadows and pastures (Tälle et al., 2016).

Climate significantly influenced the flowering phenology in a species‐specific way. Advances in FFD are frequently reported in response to climate alterations (Fitter & Fitter, 2002; Lesica & Kittelson, 2010; Menzel et al., 2006; Miller‐Rushing & Primack, 2008). In our study, advances in FFD might be mostly related to increased spring precipitation (Dorji et al., 2020) and increased minimum temperatures (Appendix S1, Figure S11) under future climate conditions that might enhance plant growth and thus phenology. Advances in LFD, on the other hand, might be mainly explained by the reduced precipitation in the future climate plots during the summer months (June‐Sept, Appendix S1, Figures S1, S12) that coincide with the main flowering time of many species (Figure 1, Appendix S1, Figure S3). Drought stress can lead to a trade‐off between reproduction and survival so that plants might shift their priorities to survival, ending flowering earlier (Galen, 2000; Lauder et al., 2019). However, due to the application of a whole scenario of future climate, the effects of altered precipitation patterns and altered temperature cannot be disentangled.

An important aim of this study was to test whether climate and land use interactively affect the flowering phenology of grassland species. We did not find statistical evidence for the interacting effects of climate and land use for the flowering phenology of the investigated grassland species, that is, phenological responses to climate did not differ between land‐use types. However, monitoring of the microclimate and ‐site conditions indicated that the climate treatment differently affected microclimate and ‐site conditions on meadows and pastures (Appendix S1, Figures S9–S12). We observed that under future climate pastures were warmer (Appendix S1, Figure S11) and drier (especially in deeper soil layers, Appendix S1, Figure S12) and showed larger proportions of open soil in summer (Appendix S1, Figure S9) compared to meadows that in contrast had slightly higher litter cover under future climate (Appendix S1, Figure S10). Furthermore, competition may play a greater role on meadows than on pastures because of the higher stand density that mediates climate change effects differently (Bütof et al., 2012; Tälle et al., 2016). Thus, we conclude that growing conditions on meadows and pastures were modified by the future climate in different ways, making interactive effects of climate and land use on phenology or other variables like survival or productivity likely. However, the lack of those interactions in our study could be related to the fact that microclimatic differences were not strong enough to influence phenology in a detectable way, as well as high variability of the data.

The species‐specific responses in our study and across other studies show that the drivers of flowering phenology are complex and might, depending on the context, be driven by various factors such as temperature, soil moisture, accumulated heat, or biotic interactions (Bock et al., 2014; Pau et al., 2011). We encourage further studies investigating each of these effects separately to contribute to a better understanding on species‐specific responses to land use and climate.

### 4.2 Explaining phenological shifts by functional traits

Functional traits explained species‐specific phenological shifts in response to climate as has also been shown in previous studies (Bucher et al., 2018; Bucher & Römermann, 2020; König et al., 2018), but trait–response relationships did not differ between land‐use types.

Plant species with an early phenological niche (early flowering) have been frequently reported to advance their phenology more strongly in response to changing climate compared to late flowering species (e.g., Fitter & Fitter, 2002; Lesica & Kittelson, 2010; Menzel et al., 2006; Miller‐Rushing & Primack, 2008; Rauschkolb et al., 2024). In contrast, in our study, we found that late flowering species showed the strongest advances in FFD and LFD, also reported by Bucher et al. (2018) for FFD. Phenological advances can relate to the escape from drought conditions (Franks et al., 2007) and drought can lead to phenological advances of the same magnitude as one decade of gradual warming (Jentsch et al., 2009). Moreover, drought stress can lead to a trade‐off between reproduction and survival, leading plants to prioritize survival and consequently end flowering earlier (Galen, 2000; Lauder et al., 2019). As in our experimental setup summer precipitation was drastically reduced under future climate conditions (Appendix S1, Figure S1), species with a later phenological niche (i.e., flowering in summer) may therefore be more affected by drought and subsequently advanced flowering start and end. However, in our study, we might have missed a few very early flowering species, as we started the phenological monitoring in the beginning of April. We thus recommend, when working in comparable grassland systems, to start phenological monitoring earlier, if possible.

Long flowering species (length of phenological niche) ended their flowering earlier and thus shortened their flowering under future climate. In contrast to short flowering species, they face a higher probability that the timing of land management coincides with the flowering period, damaging vegetative and reproductive parts of the plant. Bucher and Römermann (2020) found similar patterns along an elevational gradient, where land use did not play a prominent role. Furthermore, long flowering species are more likely to flower during high summer, when drought conditions are most pronounced under future climate and are thus more likely to end flowering earlier.

Species with a high SLA ended flowering earlier under future climate consequently shortening their flowering durations more strongly. Plants with a high SLA have thinner and less resistant leaves and might thus face more damage by mowing or grazing and are also less resistant to drought (Díaz et al., 2016; Reich et al., 1997; Wright et al., 2004). Species with a high SLA follow an acquisitive strategy (Díaz et al., 2016) that may allow a more plastic response to climate, but also a stronger need to escape drought conditions (Blumenthal et al., 2020; Griffin‐Nolan et al., 2019; Visakorpi et al., 2023; Zhang et al., 2020). König et al. (2018) also found stronger phenological shifts with increasing SLA on a global scale while Bucher et al. (2018) found an opposing relationship on the local scale along an elevational gradient. Interestingly, species with a high mowing tolerance less strongly advanced their flowering but shortened their flowering duration more strongly under future climate. Mowing tolerance is closely related to regeneration capacity, growth rate and the ability to store sufficient assimilates prior to mowing (Briemle et al., 2002). Thus, mowing tolerance is, just like SLA, ecologically related to the growth strategy (conservative vs. acquisitive) but no correlation between SLA and mowing tolerance was found in our dataset (Appendix S1, Figure S2). Additionally, a high forage value was related to stronger advances of LFD under future climate. As the forage value for livestock strongly relates to the plants' protein and mineral content as well as the growth rate (Briemle et al., 2002), a high forage value can also be associated with an acquisitive strategy.

Trampling tolerance that is related to plant height, growth, and life form (Briemle et al., 2002) as well as the trait plant height did not play a role in mediating phenological climate responses in our study. Although plant height did not relate to phenological shifts in our models (but see Appendix S1, Table S2), we suggest that it should still be considered in future studies as it was frequently shown to be of great importance in explaining phenological patterns and shifts (Huang et al., 2018; König et al., 2018; Sporbert et al., 2022; Zhu et al., 2016).

To summarize, our results indicate that late flowering species have stronger advanced flowering start and end, which is likely related to drought escape and survival over reproduction. Further, our results show that long flowering species and species with an acquisitive strategy (high SLA, mowing tolerance, and forage value), which are more susceptible to stressful conditions like summer drought (Díaz et al., 2016) were more strongly affected by future climate, advancing flowering end and consequently shortening flowering duration. Drought‐related decreases in flowering durations were observed before (Llorens & Peñuelas, 2005; Steyn et al., 1996), but contrasting responses were found as well (Jentsch et al., 2009; Llorens & Peñuelas, 2005). In contrast, early and short flowering species are less likely to be affected by land management and summer droughts and species following a more conservative strategy have a higher drought resistance accompanied by a lower phenotypic plasticity (Blumenthal et al., 2020; Griffin‐Nolan et al., 2019; Visakorpi et al., 2023; Zhang et al., 2023). Thus, these species did not show strong responses. Another reason for species not responding to climate and/or land‐use treatments may relate to the comparably short duration of the experiment (6 years), that makes genetic differentiation or evolutionary processes rather unlikely in our study system (not impossible though: Bucharova et al., 2024; Franks et al., 2007; Rauschkolb et al., 2022; Zopfi, 1993). Non‐responding species could also be rather controlled by photoperiod than by climate (Flynn & Wolkovich, 2018; Meng et al., 2021) or characterized by a generally lower trait plasticity (Zhang et al., 2020), but further investigations would be necessary to test this.

Overall, traits related to growth rate and competitive ability, but also the phenological niche (FFD, FD) were important traits mediating climate‐driven phenological responses. In our study system, namely semi‐natural and extensively managed grasslands, traits like forage value and mowing tolerance seem promising to explain differing climate responses among species and potentially land‐use types and can complement “classical” functional traits.

We did not find evidence that traits differently affected phenological climate responses on meadows compared to pastures. This was not expected, as depending on the land‐use type different traits were expected to be advantageous to cope with the different disturbances and microsite conditions on meadows and pastures as outlined above (Zhu et al., 2016). As we used mean values from the TRY database for plant height and SLA, we did not capture the intraspecific trait variability (ITV) that we might expect for the different land‐use types and climate treatments. Thus, our study may underestimate the effect of those traits (Zhang et al., 2020). However, we were not able to measure traits in situ due to constrained sampling possibilities owing to multiple side experiments running on the plots. We would recommend measuring the respective traits in situ if possible and to add also relevant physiological traits (Bucher et al., 2018; Visakorpi et al., 2023) to account for the role of ITV.

## 5 CONCLUSION

This study contributes to the understanding on how climate change and land use impact temperate grassland systems and how functional traits can mediate those impacts. Both global change drivers, climate and land use, affected the flowering phenology in a species‐specific way, but we did not find evidence for the interacting effects of climate and land use on phenology. Still, we found that microsite conditions on meadows and pastures were differently affected by future climate, making divergent effects on plant phenology (but also plant vitality, e.g., survival or productivity) likely and should be further explored. Particularly, we recommend further research focusing on microclimatic and microsite effects on phenology and phenology–trait relationships including a larger species set and maybe more importantly, considering that also traits strongly respond to variations in the environment, suggesting the need to measure traits in situ. We further conclude that functional traits and grassland indicator values offer a promising approach to understanding phenological responses to climate, with grassland indicator values being particularly useful when focusing on different grassland management practices.

The observed phenological shifts under future compared to ambient climate and the related traits mirror a phenological escape from drought which is particularly relevant in summer. Thus, late flowering, long flowering, and acquisitive species were particularly affected and shifted and shortened their flowering while species with the opposite traits did not. Thus, our findings suggest that under future climate the community of simultaneously flowering plant species will be changed especially during summer. This may therefore lead to a shortage of available pollinator resources (pollen and nectar) during summer, affecting pollinator fitness and pollination alike. To better be able to understand potential implications for pollinators within this experiment, the flowering intensity, flower cover as well as nectar and pollen characteristics should be considered in future studies.

## AUTHOR CONTRIBUTIONS

**Carolin Plos:** Conceptualization (equal); formal analysis (lead); investigation (lead); methodology (equal); visualization (lead); writing – original draft (lead); writing – review and editing (equal). **Isabell Hensen:** Conceptualization (equal); methodology (equal); resources (lead); writing – review and editing (equal). **Lotte Korell:** Conceptualization (supporting); formal analysis (supporting); methodology (supporting); writing – review and editing (equal). **Harald Auge:** Conceptualization (supporting); formal analysis (supporting); methodology (supporting); writing – review and editing (equal). **Christine Römermann:** Conceptualization (equal); methodology (equal); resources (equal); writing – review and editing (equal).

## CONFLICT OF INTEREST STATEMENT

The authors declare no conflict of interest.

## Supporting information


Data S1.

## Data Availability

Link to the figshareproject: https://figshare.com/projects/Plant_species_phenology_differs_between_climate_and_land‐use_scenarios_and_relates_to_plant_functional_traits/188952. Datasets (main manuscript): https://doi.org/10.6084/m9.figshare.24787530. Datasets (Appendix figures): https://doi.org/10.6084/m9.figshare.24799248.v1. R code (analysis and figures): https://doi.org/10.6084/m9.figshare.24799275.v1.
