# Supplementary material for: Plant species phenology differs between climate and land‐use scenarios and relates to plant functional traits
Source: Ecol Evol. 2024 May 23;14(5):e11441. doi: 10.1002/ece3.11441 (PMC11116844; doi:10.1002/ece3.11441)
Supplement: Supplementary file 1 — Data S1. [file ECE3-14-e11441-s001.pdf]

Appendix

Manuscript title: Plant species phenology differs between climate and land-use scenarios and relates to plant-functional traits

Authors: Carolin Plos, Isabell Hensen, Lotte Korell, Harald Auge, Christine Römermann

Journal: Ecology and Evolution

Appendix S1 – Figures

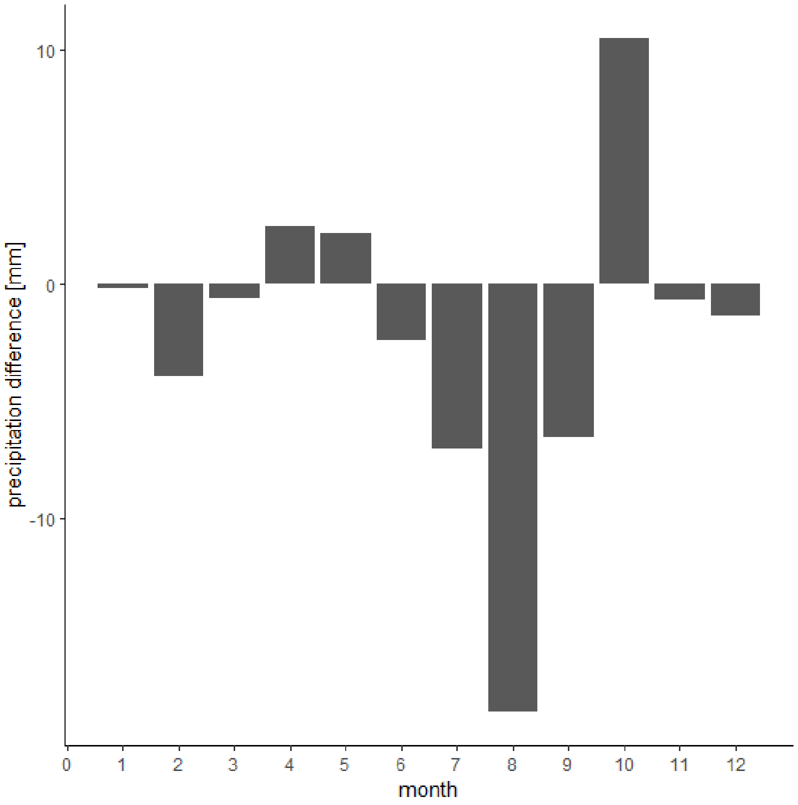

Figure S1: Realized precipitation difference in mm of future plots compared to ambient plots, summarized for each month across the study year 2020.

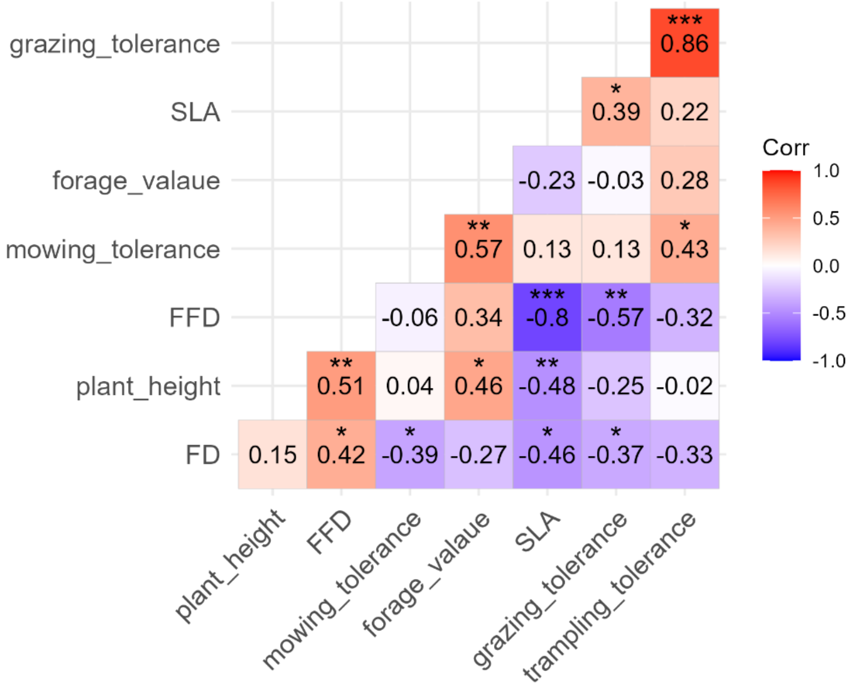

Figure S2: Correlation matrix of functional traits, based on Pearson correlations, including the correlation coefficients (*r*) and significance levels (* $p < 0.05$, ** $p < 0.01$, *** $p < 0.001$) of all pairwise combinations of functional traits. Abbreviations: SLA – specific leaf area, FFD – timing of phenological niche measured as first flowering day, FD – length of phenological niche measured as flowering duration in days.

Figure S3: Flowering times and timing of land management (mowing or grazing) across all treatments. Strips summarize the mean FFD, LFD and day of peak flowering per treatment, the x-axis shows day of the year (doy). Please note that five species (*A. millefolium, D. carthusianorum, G. album, G. verum, S. ochroleuca*) are already presented in Figure 1 in the main text and are thus not presented here.

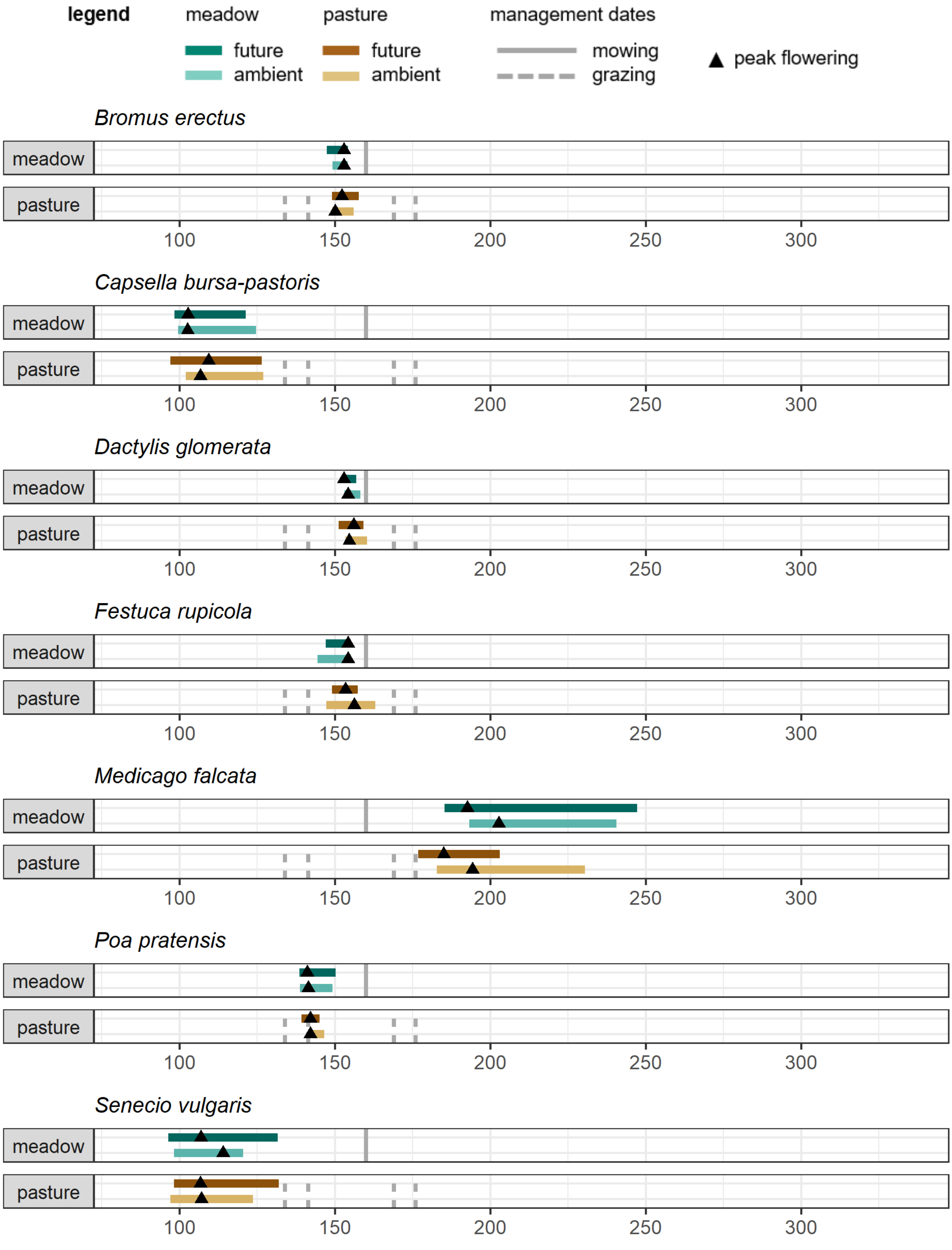

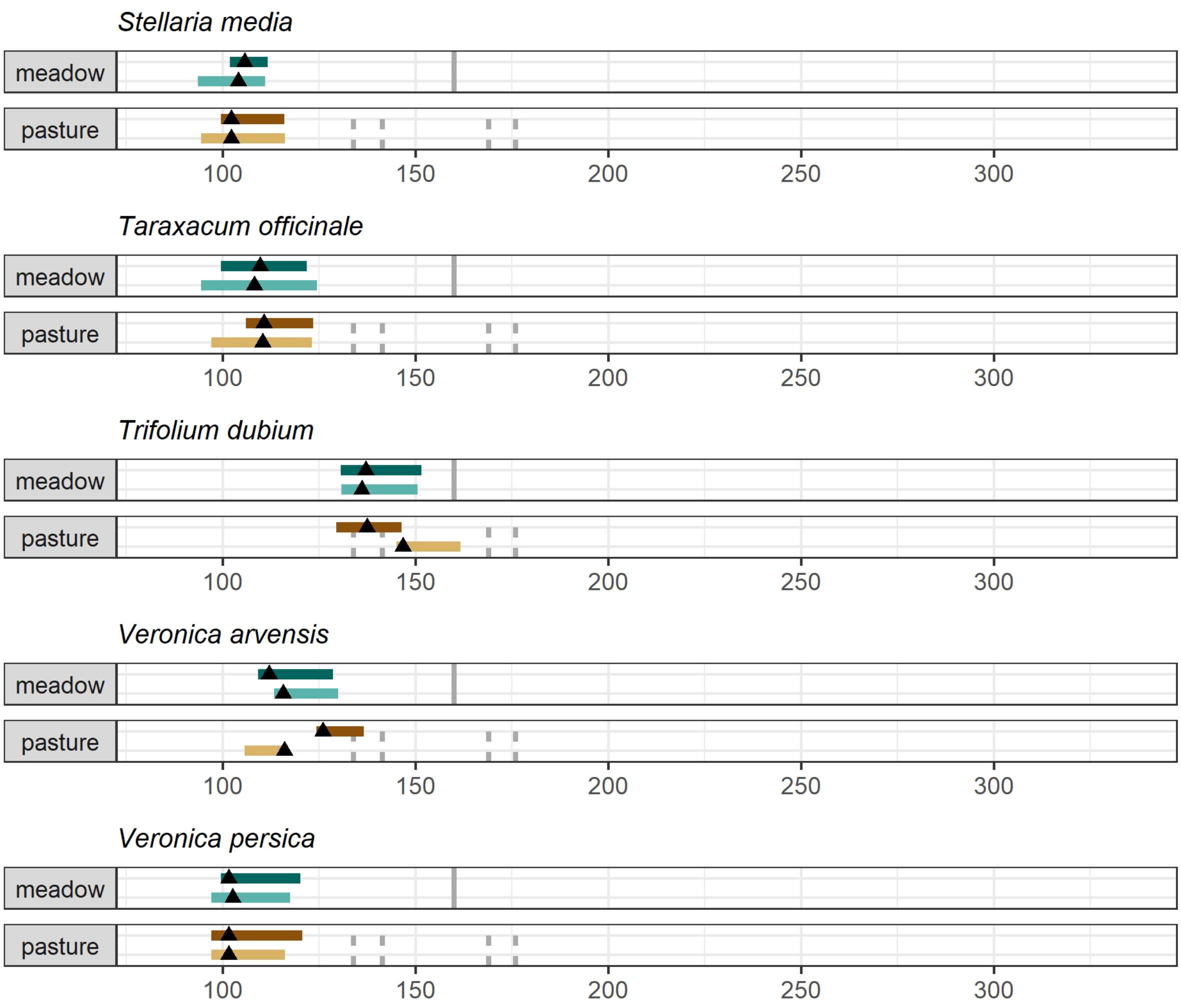

Stellaria media
meadow
pasture
100
150
200
250
300
Taraxacum officinale
meadow
pasture
100
150
200
250
300
Trifolium dubium
meadow
pasture
100
150
200
250
300
Veronica arvensis
meadow
pasture
100
150
200
250
300
Veronica persica
meadow
pasture
100
150
200
250
300

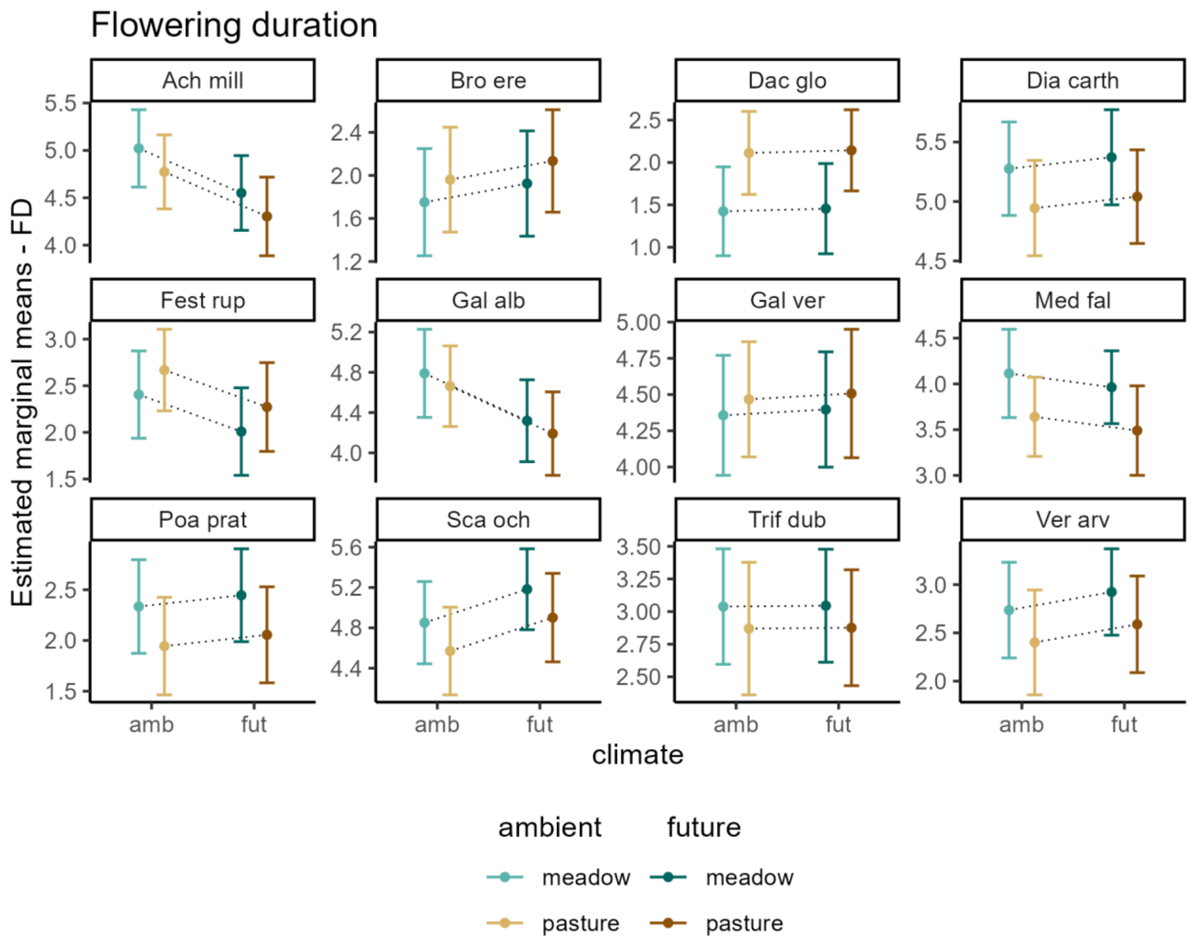

Figure S4: Effects of climate and land-use on the flowering duration. Shown are estimated marginal means of peak flowering with the 95% confidence intervals, revealed from the generalized linear mixed model. Results compare the flowering duration between ambient and future climate on meadows and pastures for each species. Letters indicate significant differences between treatments. If no letters are shown, no significant difference between treatment groups was found. Dotted lines are only shown for better interpretation of interactive effects of climate and land-use.

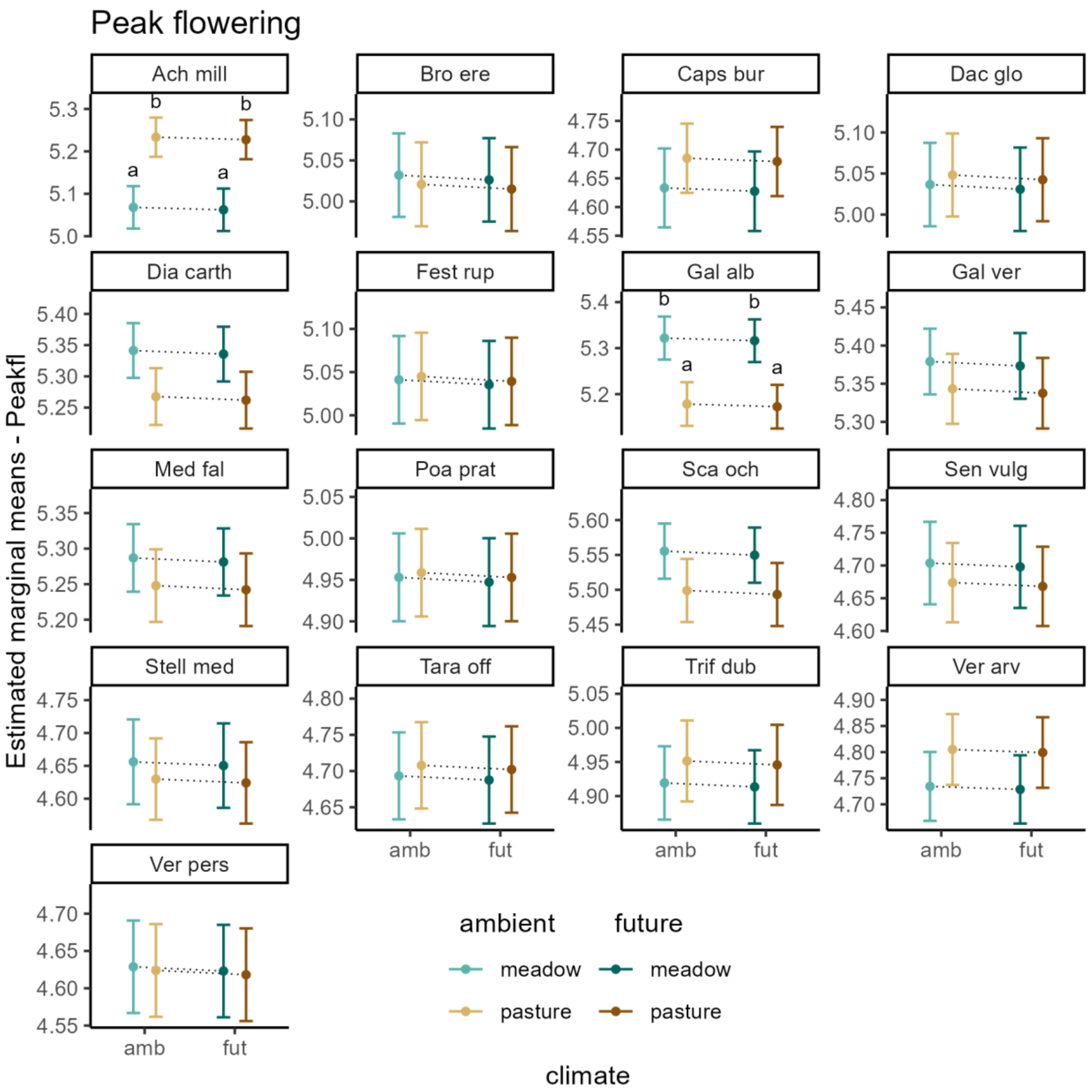

Figure S5: Effects of climate and land-use on the day of peak flowering. Shown are estimated marginal means of peak flowering with the 95% confidence intervals, revealed from the generalized linear mixed model. Results compare the day of peak flowering between ambient and future climate on meadows and pastures for each species. Letters indicate significant differences between treatments. If no letters are shown, no significant difference between treatment groups was found. Dotted lines are only shown for better interpretation of interactive effects of climate and land-use.

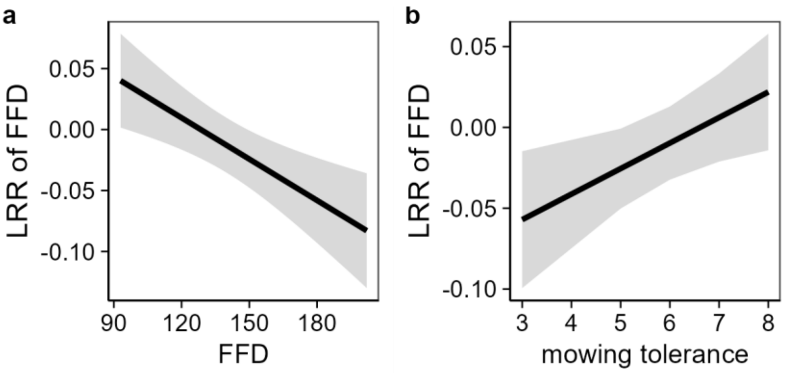

Figure S6: Relationships between traits and the shift in first flowering day due to future climate (Log response ratio, LRR), as predicted by the linear model. See also Table 3 for model results. Traits are: mean first flowering day (from ambient plots) (FFD) and mowing tolerance derived from BiolFlor.

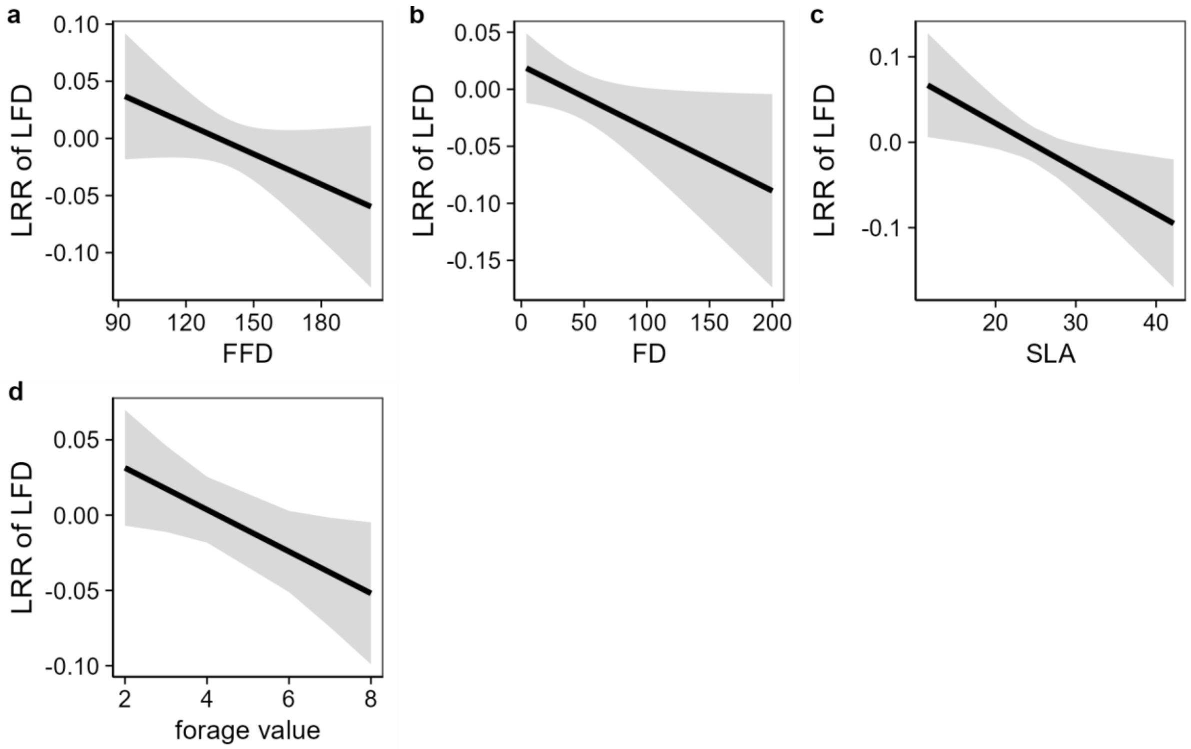

Figure S7: Relationships between traits and the shift in last flowering day (LFD) due to future climate (Log response ratio, LRR), as predicted by the linear model. See also Table 3 for model results. Traits are: mean first flowering day (from ambient plots) (FFD), mean flowering duration in days (from ambient plots) (FD), mean SLA derived from TRY (SLA) and forage value derived from BiolFlor.

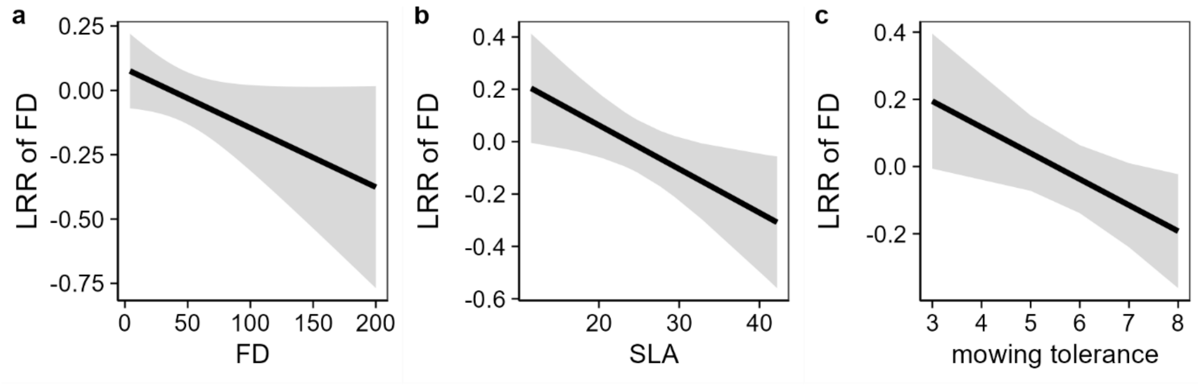

Figure S8: Relationships between traits and the shift in flowering duration (FD) due to future climate (Log response ratio, LRR), as predicted by the linear model. See also Table 3 for model results. Traits are: mean flowering duration in days (from ambient plots) (FD), mean SLA derived from TRY (SLA) and mowing tolerance derived from BiolFlor.

The coverage of open soil (%) and litter (%) was estimated weekly for each permanent plot (3m x 3m), across the monitoring season of 2020. Different proportions of litter cover and open soil were observed across land-use types and climate treatments, with pastures generally having larger proportions of open soil that remarkably increased under future climate conditions after the second grazing event in summer while on meadows the proportion of open soil was generally smaller and tended to decrease under future climate (Figure S9). In contrast, litter cover on meadows tended to increase more strongly under future climate than observed for pastures (Figure S10), although the patterns were more comparable between the land-use types.

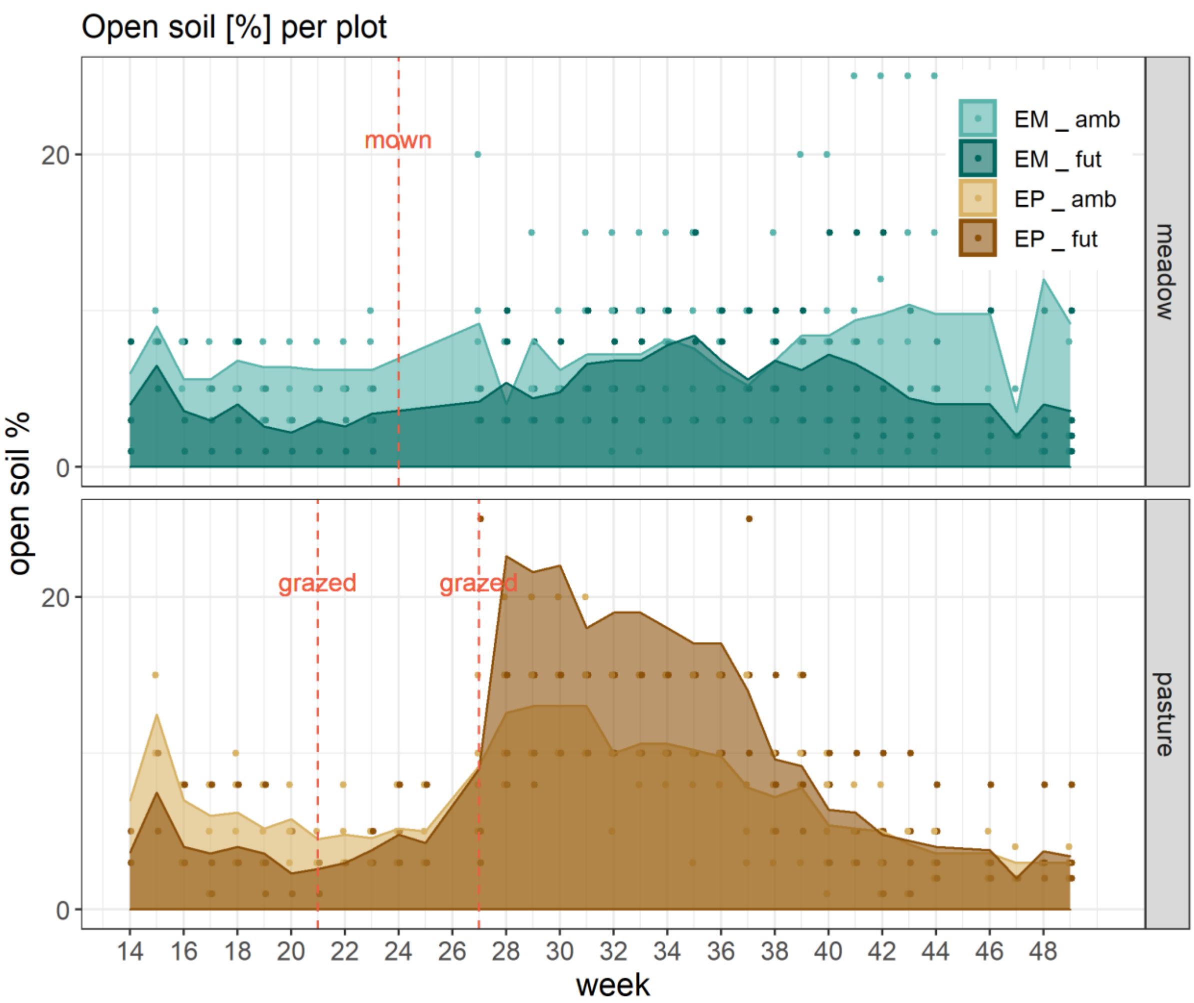

Figure S9: Percentage open soil cover per plot and timing of land management throughout the monitoring season in 2020 compared between meadows (EM) and pastures (EP) under ambient and future climate. Dots indicate the weekly open soil cover per plot, the ribbons indicate the mean weekly open soil cover across plots of the same treatment. Dashed vertical lines indicate the timing of the land use events (i.e. mowing or grazing). First grazing occurred in week 21 and 22 respectively, and second grazing in week 27 and 28 respectively, as every plot (n = 10) was grazed for 24 hours.

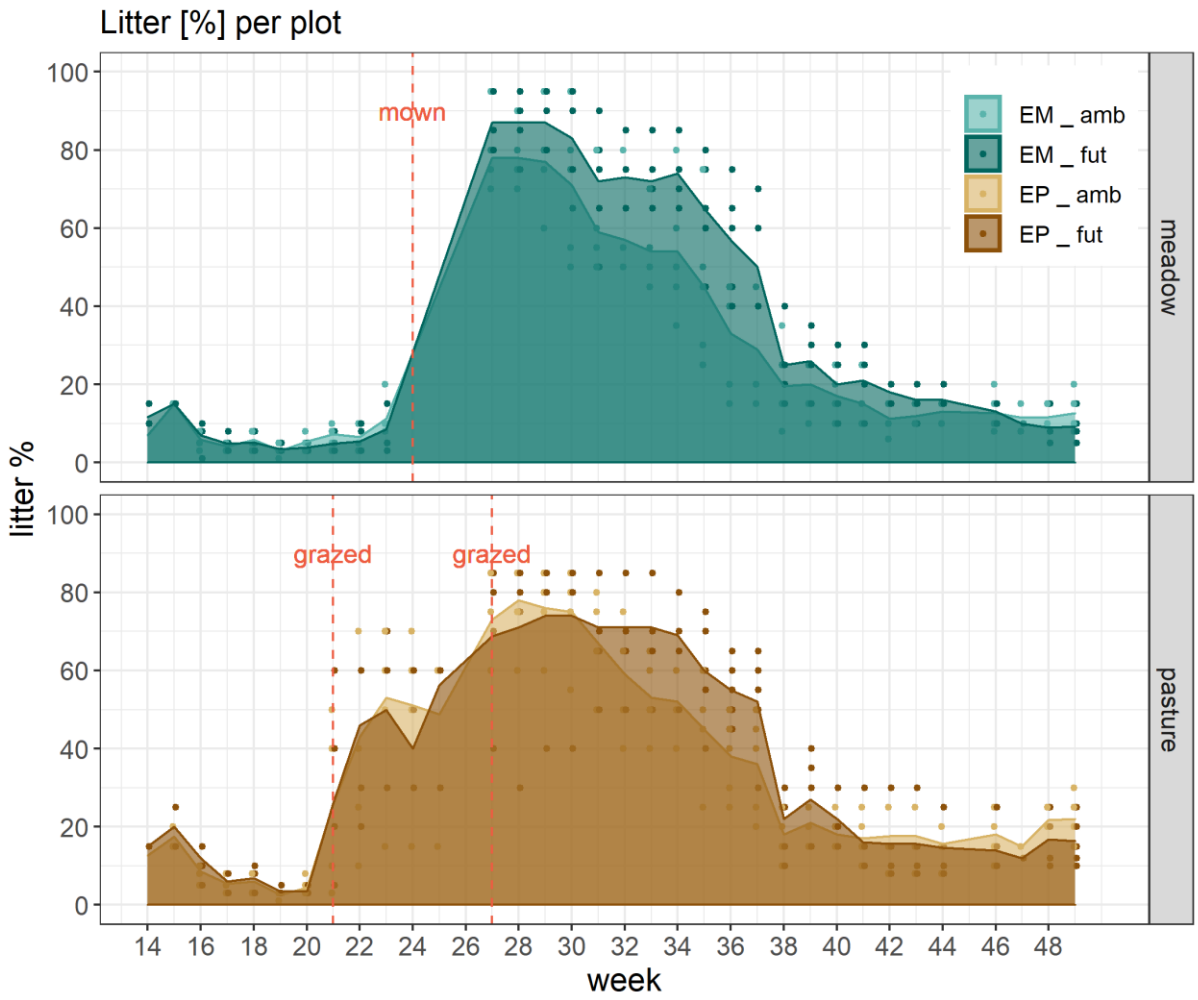

Figure S10: Percentage litter cover per plot and timing of land management throughout the monitoring season compared between meadows (EM) and pastures (EP) under ambient and future climate. Dots indicate the weekly litter cover per plot, the ribbons indicate the mean weekly litter cover. Dashed vertical lines indicate the timing of the land use events (i.e. mowing or grazing). First grazing stretched through week 21 and 22 respectively, and second grazing through week 27 and 28 respectively, as every plot (n = 10) was grazed for 24 hours.

Temperature and soil moisture were measured daily within each plot. Details on temperature and soil moisture measurements can be found in Schädler et al. (2019) and in Appendix S5 of the respective publication. Figure S11 shows that especially in the summer months the daily minimum temperature was higher under future climate regardless of land-use (a), while daily maximum temperature was generally higher in pastures (b), overall increasing mean daily temperatures on future pastures (c). Soil moisture was lower under future climate and lowest on future pastures with this effect increasing with soil depth (Figure S12).

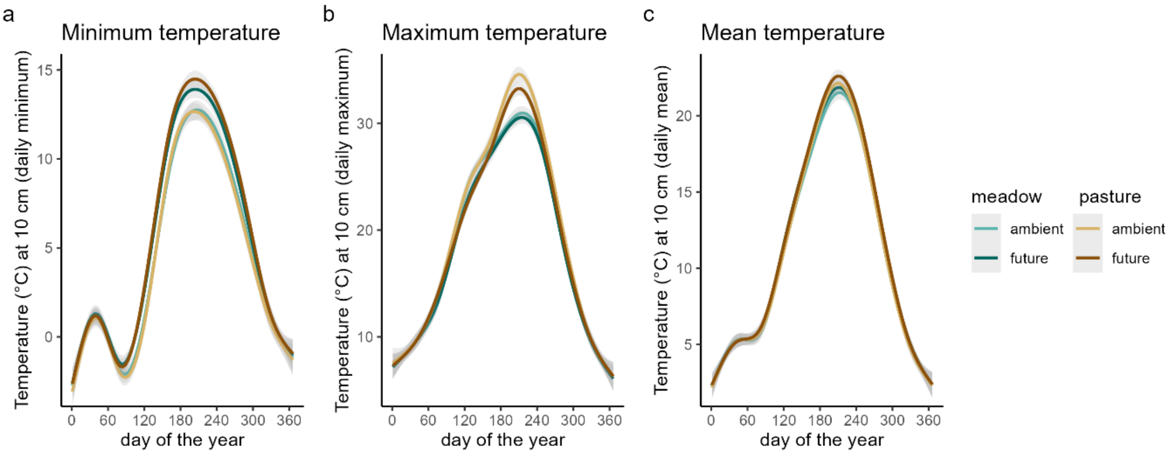

Figure S11: Daily temperatures a) minimum, b) maximum, c) mean, measured 10 cm above the ground across all meadow and pasture plots under ambient and future climate for 2020.

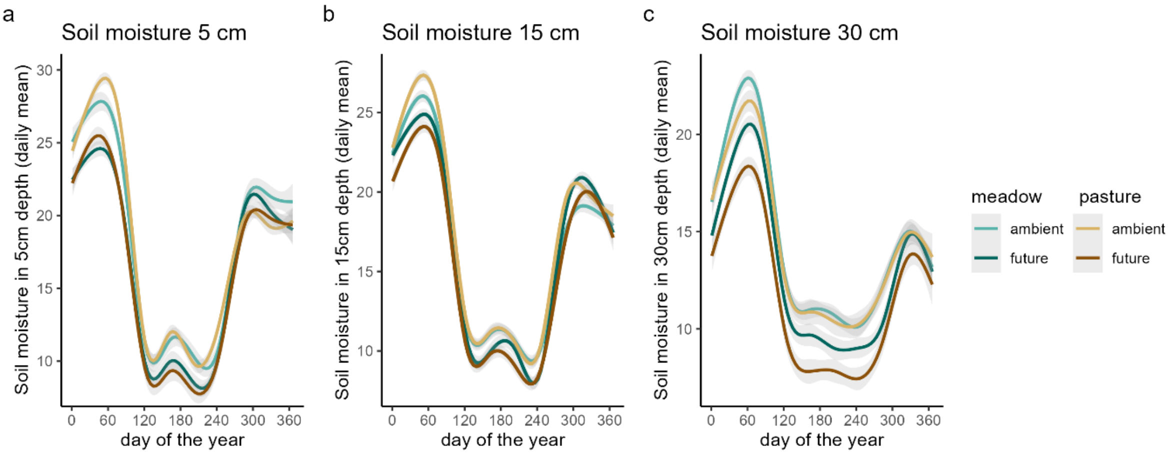

Figure S12: Mean daily soil moisture measured in a) 5 cm, b) 15 cm and c) 30 cm depth across all meadow and pasture plots under ambient and future climate for 2020.

Appendix S2 – Tables

Table S1: Model selection table explaining shifts in first flowering day (FFD shift) to future climate by functional traits, grassland indicator values and land-use type. The table shows the best models according to AICc with a deltaAIC < 2 derived by the *dredge* function (*MuMIn* package, Bartoń, 2023). Numbers (i.e. estimates) indicate traits that were included in the final models. Empty cells refer to traits that were excluded from the models.

Intercept	land use	forage value	FD	FFD	height	SLA	mowing tolerance	trampling tolerance	df	logLik	AICc	delta	weight
-0.012				-0.038			0.028		4	43.41	-77.2	0	0.234
-0.012				-0.044			0.036	-0.020	5	44.79	-77.1	0.13	0.218
-0.012				-0.053	0.014		0.036	-0.022	6	45.55	-75.4	1.78	0.096

Table S2: Model selection table explaining shifts in last flowering day (LFD shift) to future climate by testing functional traits, grassland indicator values and land-use type. The table shows the best models according to AICc with a deltaAIC < 2 derived by the *dredge* function (*MuMIn* package, Bartoń, 2023). Numbers (i.e. estimates) indicate traits that were included in the final models. Empty cells refer to traits that were excluded from the models.

Intercept	land use	forage value	FD	FFD	height	SLA	mowing tolerance	land use : FFD	land use : SLA	df	logLik	AICc	delta	weight
-0.005		-0.028	-0.027	-0.030		-0.045				6	47.41	-79.2	0	0.096
-0.005		-0.035	-0.032			-0.024				5	45.83	-79.2	0.02	0.095
0.004	+			0.001	-0.021	-0.017		+	+	8	50.85	-78.8	0.34	0.081
-0.005				-0.039	-0.021	-0.043				5	45.49	-78.5	0.7	0.068
0.004	+			-0.005		-0.012		+	+	7	48.73	-78.4	0.8	0.064
-0.005				-0.04699		-0.0392				4	43.90	-78.2	0.97	0.059
-0.005		-0.028	-0.031		-0.018	-0.031				6	46.92	-78.2	1	0.058
-0.005		-0.026	-0.019							4	43.86	-78.1	1.05	0.057
0.005	+	-0.027	-0.026	-0.013		-0.046		+		8	50.37	-77.9	1.3	0.05
-0.005		-0.021								3	42.36	-77.8	1.38	0.048
0.003	+			-0.021	-0.021	-0.046		+		7	48.39	-77.7	1.49	0.046
-0.005					-0.020					3	42.29	-77.7	1.53	0.045
-0.005		-0.022	-0.026	-0.028	-0.015	-0.049				7	48.30	-77.5	1.66	0.042
-0.005			-0.016	-0.036	-0.023	-0.049				6	46.54	-77.4	1.74	0.04
-0.005		-0.016		-0.040		-0.037				5	44.95	-77.4	1.77	0.04

0.003	+			-0.028		-0.041		+		6	46.51	-77.4	1.82	0.039
0.004	+	-0.015		0.0003		-0.011		+	+	8	50.03	-77.2	1.97	0.036
-0.005			-0.023	-0.033	-0.022	-0.047	-0.018			7	48.14	-77.2	1.98	0.036

Table S3: Model selection table explaining shifts in flowering duration (FD shift) to future climate by testing functional traits, grassland indicator values and land-use type. The table shows the best models according to AICc with a deltaAIC < 2 derived by the *dredge* function (*MuMIn* package, Bartoń, 2023). Numbers (i.e. estimates) indicate traits that were included in the final models. Empty cells refer to traits that were excluded from the models.

Intercept	land use	forage value	FD	FFD	SLA	mowing tolerance	trampling tolerance	df	logLik	AICc	delta	weight
-0.022			-0.115		-0.142	-0.138		5	-0.82	14.1	0	0.203
-0.022			-0.105		-0.151	-0.165	0.076	6	0.25	15.1	0.99	0.124
-0.022					-0.095	-0.099		4	-2.80	15.2	1.07	0.119
-0.022			-0.104	-0.1072	-0.225	-0.129		6	0.13	15.4	1.24	0.109
-0.022					-0.109	-0.135	0.088	5	-1.48	15.5	1.32	0.105
-0.022				-0.1281	-0.197	-0.093		5	-1.57	15.6	1.5	0.096
-0.022						-0.112		3	-4.51	16	1.81	0.082
0.029	+		-0.119		-0.144	-0.139		6	-0.17	16	1.84	0.081
-0.022		-0.125	-0.116		-0.190			5	-1.75	16	1.85	0.08

Table S4: Model selection table explaining shifts in peak flowering (Peakfl shift) to future climate by testing functional traits, grassland indicator values and land-use type. The table shows the best models according to AICc with a deltaAIC < 2 derived by the *dredge* function (*MuMIn* package, Bartoń, 2023). Numbers (i.e. estimates) indicate traits that were included in the final models. Empty cells refer to traits that were excluded from the models.

Intercept	land use	forage value	FD	FFD	height	SLA	mowing tolerance	trampling tolerance	df	logLik	AICc	delta	weight
-0.002									2	49.13	-93.8	0	0.232
-0.011	+								3	49.69	-92.5	1.35	0.118
-0.002								0.008	3	49.65	-92.4	1.44	0.113
-0.002		-0.008							3	49.59	-92.3	1.55	0.107
